# Supplementary material for: Resolving the full spectrum of human genome variation using Linked-Reads
Source: Genome Res. 2019 Apr;29(4):635–45. doi: 10.1101/gr.234443.118 (PMC6442396; doi:10.1101/gr.234443.118)
Supplement: Supplemental Material [file supp_gr.234443.118_Supplemental_longranger-2.2.zip › longranger-2.2.tar.bz2!/lr2.2-public/lib/go/src/code.google.com/vitess/docs/user-guide/transport-security-model.html]

Vitess / Transport Security Model


Toggle navigation

- Overview
- Guides
- Reference
- Blog
- GitHub

- #### Overview

  - What is Vitess
  - Scaling MySQL with Vitess
  - Key Concepts
- #### Getting Started

  - Run Vitess on Kubernetes
    - Custom Docker Build
  - Run Vitess Locally
- #### User Guide

  - Introduction- Client Libraries- Backing Up Data- Reparenting
        - Schema Management
        - VSchema Guide
        - Schema Swap (Tutorial)
        - Sharding
          - Horizontal Sharding (Tutorial, automated)
          - Horizontal Sharding (Tutorial, manual)
          - Sharding in Kubernetes (Tutorial, automated)
          - Sharding in Kubernetes (Tutorial, manual)
        - Vitess Sequences
        - MySQL Server Protocol
        - Vitess and Replication
        - Update Stream
        - Row Based Replication
        - Topology Service
        - Transport Security Model
        - Launching
          - Scalability Philosophy
          - Production Planning
          - Server Configuration
          - 2PC Guide
          - Troubleshooting
        - Upgrading
- #### Reference Guides

  - Vitess API- vtctl Commands
- #### Other Resources

  - Presentations- Blog- Roadmap
- #### Contributing

  - Contributing to Vitess- GitHub Workflow- Code Reviews
- GitHub

# Transport Security Model

- #### Overview

  - What is Vitess
  - Scaling MySQL with Vitess
  - Key Concepts
- #### Getting Started

  - Run Vitess on Kubernetes
    - Custom Docker Build
  - Run Vitess Locally
- #### User Guide

  - Introduction- Client Libraries- Backing Up Data- Reparenting
        - Schema Management
        - VSchema Guide
        - Schema Swap (Tutorial)
        - Sharding
          - Horizontal Sharding (Tutorial, automated)
          - Horizontal Sharding (Tutorial, manual)
          - Sharding in Kubernetes (Tutorial, automated)
          - Sharding in Kubernetes (Tutorial, manual)
        - Vitess Sequences
        - MySQL Server Protocol
        - Vitess and Replication
        - Update Stream
        - Row Based Replication
        - Topology Service
        - Transport Security Model
        - Launching
          - Scalability Philosophy
          - Production Planning
          - Server Configuration
          - 2PC Guide
          - Troubleshooting
        - Upgrading
- #### Reference Guides

  - Vitess API- vtctl Commands
- #### Other Resources

  - Presentations- Blog- Roadmap
- #### Contributing

  - Contributing to Vitess- GitHub Workflow- Code Reviews

# Vitess Transport Security Model

Vitess exposes a few RPC services, and internally also uses RPCs. These RPCs
may use secure transport options. This document explains how to use these
features.

## Overview

The following diagram represents all the RPCs we use in a Vitess cluster:

There are two main categories:

- Internal RPCs: they are used to connect Vitess components.
- Externally visible RPCs: they are use by the app to talk to Vitess.

A few features in the Vitess ecosystem depend on authentication, like Called ID
and table ACLs. We'll explore the Caller ID feature first.

The encryption and authentication scheme used depends on the transport
used. With gRPC (the default for Vitess), TLS can be used to secure both
internal and external RPCs. We'll detail what the options are.

## Caller ID

Caller ID is a feature provided by the Vitess stack to identify the source of
queries. There are two different Caller IDs:

- **Immediate Caller ID**: It represents the secure client identity when it
  enters the Vitess side:
  - It is a single string, represents the user connecting to Vitess (vtgate).
  - It is authenticated by the transport layer used.
  - It is used by the Vitess TableACL feature.
- **Effective Caller ID**: It provides detailed information on who the
  individual caller process is:
  - It contains more information about the caller: principal, component,
    sub-component.
  - It is provided by the application layer.
  - It is not authenticated.
  - It is exposed in query logs to be able to debug the source of a slow query,
    for instance.

## gRPC Transport

### gRPC Encrypted Transport

When using gRPC transport, Vitess can use the usual TLS security features
(familiarity with SSL / TLS is necessary here):

- Any Vitess server can be configured to use TLS with the following
  command line parameters:
  - grpc\_cert, grpc\_key: server cert and key to use.
  - grpc\_ca (optional): client cert chains to trust. If specified, the client
    must use a certificate signed by one ca in the provided file.
- A Vitess go client can be configured with symetrical parameters to enable TLS:
  - ...\_grpc\_ca: list of server cert signers to trust.
  - ...\_grpc\_server\_name: name of the server cert to trust, instead of the
    hostname used to connect.
  - ...\_grpc\_cert, ...\_grpc\_key: client side cert and key to use (when the
    server requires client authentication)
- Other clients can take similar parameters, in various ways, see each client
  for more information.

With these options, it is possible to use TLS-secured connections for all parts
of the system. This enables the server side to authenticate the client, and / or
the client to authenticate the server.

Note this is not enabled by default, as usually the different Vitess servers
will run on a private network (in a Cloud environment, usually all local traffic
is already secured over a VPN, for instance).

### Certificates and Caller ID

Additionally, if a client uses a certificate to connect to Vitess (vtgate), the
common name of that certificate is passed to vttablet as the Immediate Caller
ID. It can then be used by table ACLs, to grant read, write or admin access to
individual tables. This should be used if different clients should have
different access to Vitess tables.

### Caller ID Override

In a private network, where SSL security is not required, it might still be
desirable to use table ACLs as a safety mechanism to prevent a user from
accessing sensitive data. The gRPC connector provides the
grpc\_use\_effective\_callerid flag for this purpose: if specified when running
vtgate, the Effective Caller ID's principal is copied into the Immediate Caller
ID, and then used throughout the Vitess stack.

**Important**: this is not secure. Any user code can provide any value for
the Effective Caller ID's principal, and therefore access any data. This is
intended as a safety feature to make sure some applications do not misbehave.
Therefore, this flag is not enabled by default.

### Example

For a concrete example, see
test/encrypted\_transport.py
in the source tree. It first sets up all the certificates, and some table ACLs,
then uses the python client to connect with SSL. It also exercises the
grpc\_use\_effective\_callerid flag, by connecting without SSL.

Contact: vitess@googlegroups.com  **·**  
Announcements  **·**  
© 2017 Vitess powered by Google Inc
